# Effect of Dietary Inclusion of Full-Fat Insect Meals (*Hermetia illucens* and *Tenebrio molitor*) for Broiler Chickens: Live Performance, Carcass Yield, Meat Quality, Blood Profiles, and Intestinal Morphometry

**DOI:** 10.3390/ani16060939

**Published:** 2026-03-17

**Authors:** Márk Tóth, Yazavinder Singh, Krisztián Balogh, Erika Zándoki, Szabina Kulcsár, Benjámin Kövesi, Zsolt Ancsin, Balázs Gregosits, Miklós Mézes, Mária Kovács-Weber, Márta Erdélyi

**Affiliations:** 1 Department of Feed Safety, Hungarian University of Agriculture and Life Sciences, Páter Károly u. 1., H-2100 Gödöllő, Hungary; balogh.krisztian.milan@uni-mate.hu (K.B.); mezes.miklos@uni-mate.hu (M.M.);; 2 Department of Animal Husbandry Technology and Animal Welfare, Hungarian University of Agriculture and Life Sciences, Páter Károly u. 1., H-2100 Gödöllő, Hungary; kovacs-weber.maria@uni-mate.hu; 3 Department of Agronomy, Food, Natural Resources, Animals and Environment (DAFNAE), University of Padova, Viale Dell’Università 16, Stecca 1, 35020 Legnaro, Padova, Italy; yazavinder.singh@unipd.it; 4 HUN-REN-MATE Mycotoxins in the Food Chain Research Group, Hungarian University of Agriculture and Life Sciences, Páter Károly u. 1., H-2100 Gödöllő, Hungary; 5 Vitafort First Hungarian Feed Production and Distribution Zrt., Szabadság u. 3., H-2370 Dabas, Hungary

**Keywords:** insect meal, broiler chickens, meat quality, blood profile, intestinal morphometry

## Abstract

The present study evaluated the effects of dietary inclusion of *Hermetia illucens* (HI) and *Tenebrio molitor* (TM) larvae meals at 2% and 4% on growth performance, carcass yield, meat quality, blood parameters, and intestinal morphometry in broiler chickens. A total of 1750 one-day-old male Ross 308 broilers were assigned to five dietary treatments: a Control diet, or diets containing 2% or 4% of HI meal (HI2, HI4) or TM meal (TM2, TM4). Growth performance, feed intake (FI), feed conversation ratio (FCR), and mortality were unaffected by dietary treatments. Breast yield increased significantly in insect-fed groups (29.2–29.9%) compared with Control (27.6%). Birds fed HI4 exhibited lower breast pH (5.77 vs. 5.89) and increased cooking loss (29.2% vs. 27.3%), suggesting reduced WHC within acceptable ranges. Thigh meat showed dose-dependent lipid accumulation in insect-fed birds. Serum total cholesterol increased in TM-fed birds with elevated HDL-cholesterol, while LDL-cholesterol remained unaffected. TM meal specifically induced shorter ileal length compared with Control and HI-fed groups. Overall, both full-fat insect meals can be safely incorporated at low inclusion levels without adverse effects on broiler growth, health, or carcass quality.

## Simple Summary

Chickens are one of the World’s most important food sources, but raising them requires large amounts of feed, which is often expensive and imported from distant countries. Researchers are searching for sustainable alternatives that are locally produced and environmentally friendly. Insects, particularly black soldier fly and yellow mealworms, are promising candidates because they can be raised on agricultural waste and food scraps, converting them into high-quality feedstuffs. This research tested whether insect-based meals could be safely used in broiler chicken diets. A total of 1750 chickens were divided into five groups: one Control group fed standard diet, and four groups fed insect meal at two different levels for six weeks. Both types of insect meals proved supported normal growth, with all chickens growing normally and remaining healthy. Blood tests revealed that chickens fed mealworm meal had modified serum lipid profiles. The main barrier to using insect feed is cost, higher than that of the conventional protein sources like soybean meal. This research demonstrates that insect-based feeds are nutritious alternatives that can be successfully incorporated into broiler diets without compromising animal health or growth.

## 1. Introduction

The global demand for poultry meat, particularly broiler chicken, has increased significantly in recent years due to its nutritional value and popularity among consumers [1]. As the poultry industry seeks to meet this demand, ensuring a stable, cost-effective feed supply remains critical. To address this, researchers and industry professionals are actively investigating alternative protein sources that are economically viable and environmentally sustainable. Options such as rapeseed meal [2], camelina meal [3], corn gluten meal [4] and high-protein dried distillers’ grains with solubles [5,6] have been explored, as substitutes for soybean meal, which is the primary source of protein in poultry diets. More recently, insect-based protein sources have gained significant attention as a promising and innovative approach to poultry nutrition [7,8].

The use of insect meals in poultry feed has been studied for several species, including HI and TM, due to their favorable nutrient composition and potential to reduce dependence on conventional protein sources [9,10]. In addition to their nutritional value, insect rearing systems promote sustainability by utilizing agro-industrial by-products, thus supporting circular economy strategies [11].

These insects have a valuable nutritional profile. They provide high levels of protein with a balanced composition of amino acids [12], and high levels of lipids. The most common fatty acids (FAs) found in various species are oleic acid (C18:1), linoleic acid (C18:2), palmitic acid (C16:0), lauric acid (C12:0), and stearic acid (C18:0) [13,14]. Medium chain fatty acids (MCFAs), which are abundant in insect lipids, have been shown to possess antimicrobial properties [13,14]. Furthermore, insects have been reported to contain antioxidants and bioactive compounds, including phenolic compounds and pigments, which could enhance meat quality and promote bird health [15,16]. Their chitin content has been reported to exert prebiotic effects promoting beneficial cecal microbiota such as *Faecalibacterium prausnitzii* and *Roseburia faecis*. These bacteria enhance the production of short-chain fatty acids (SCFAs), particularly butyrate, which serves as the primary energy source for enterocytes [17]. Furthermore, antimicrobial peptides derived from insects provide additional functional benefits by exhibiting antibacterial, antifungal, and antiviral activities [18].

Despite their promising nutritional and functional properties, the large-scale use of insect meals in poultry farming remains limited, primarily due to their high cost. At the moment, insect meals are three to four times expensive than fishmeal, and up to ten times more expensive than soybean meal [19]. This economic disparity significantly impedes commercial adoption. Previous studies have shown that, at current market prices, the inclusion of insect meal becomes economically marginal unless it can deliver substantial improvements in product quality or health outcomes [19,20]. This economic disparity supports the prevailing view in the literature that insect-derived proteins are not yet cost-competitive with traditional protein sources [21]. Elevated production costs and limited output volumes continue to constrain scalability [22]. These factors represent a significant barrier to the wider use of insect meals in commercial livestock [20,23].

Therefore, evaluating the potential of the low-dose inclusion of insect meals is particularly relevant. If measurable benefits can be achieved at levels as low as 2–4%, insect meals could be a viable alternative protein source for poultry production, balancing sustainability and economic feasibility. Direct comparisons of two insect meals under identical experimental conditions are limited in the literature, representing an important gap in understanding of species-specific effects. The present study was conducted under semi-commercial conditions. The objective was to evaluate the effects of dietary inclusion of either full-fat HI or TM meals at 2% and 4% on growth performance, carcass yield, organ development, meat quality, intestinal morphometry, and blood parameters in broiler chickens. A further aim was to directly compare the two insect meals under identical experimental conditions, enabling a species-specific assessment of their effects. It was hypothesized that low-level inclusion (2–4%) of full-fat HI or TM meal would influence broiler growth performance, carcass yield, organ development, meat quality, blood parameters, and intestinal morphometry, and that each insect species might elicit different effects due to their distinct nutrient profiles.

## 2. Materials and Methods

### 2.1. Experimental Diets and Preparation

The experimental diets included a Control diet, and diets including either 2% or 4% full-fat HI meal (HI2 and HI4) and 2% or 4% full-fat TM meal (TM2 and TM4). All diets were formulated to be isonitrogenous and isoenergetic, based on digestible amino acids and metabolizable energy. The experimental diets were based on a commercially produced concentrate, which accounted for 85% of the total formulation and was supplied by Vitafort Plc. (Dabas, Hungary). Owing to industrial confidentiality, the detailed ingredient composition of this concentrate is not disclosed. The remaining 15% of the diet was formulated to incorporate the assigned inclusion levels of HI and TM meals while meeting the nutrient requirements for broiler chickens by Ross 308 Broiler Management Handbook [24]. The ingredient and chemical composition of the complete experimental diets are presented in Table 1 and Table 2.

### 2.2. Experimental Design and Bird Management

The experiment was carried out at the animal research facility of AgriSearch Hungary Ltd. (Pécel, Hungary). All birds were managed in accordance with the guidelines set out in European Directive 2010/63/EU on the protection of animals used for scientific purposes [25], as well as in accordance with Hungarian legal regulations (32/1999. /III. 31./ and 178/2009. /XII. 29./).

A total of 1750 one-day-old male Ross 308 broiler chicks were obtained from a commercial hatchery (Babádi Baromfikeltető Ltd., Dabas, Hungary). The chicks were vaccinated against Newcastle disease, infectious bronchitis, infectious bursal disease, and fowl pox. Upon arrival, the birds were randomly assigned to five dietary treatments with 14 replicate pens per treatment and 25 birds in each pen. Each pen (2.5 m^2^) contained an 8 cm layer of wood shavings for bedding. Feed and water were provided ad libitum. Housing conditions were in line with the recommendations set out in the Ross 308 Broiler Management Handbook [24].

### 2.3. Growth Performance

Body weights (BWs) were recorded individually at weekly intervals throughout the 42-day trial to monitor growth trends. Since the birds were not individually tagged, pen averages were used to calculate body weight gain (BWG). Feed intake (FI) was determined per pen on a weekly basis, from which the feed conversion ratio (FCR) was derived. Mortality was checked and recorded daily.

### 2.4. Slaughter and Carcass Dissection Traits

On day 42, after an overnight period of feed deprivation, 140 birds (28 per treatment and 2 per replicate) were randomly selected, weighed, and slaughtered by severing their jugular veins and carotid arteries. The carcass weight (CW) was recorded after the head, neck, shanks, abdominal fat, viscera, and feathers were removed. The breast filet, legs, gizzard, and liver were excised and weighed. Carcass and organ weights were expressed as a percentage of the weight at slaughter, whereas breast and thigh weights were expressed as a percentage of the CW.

### 2.5. Meat Quality

A total of seventy birds (14 per treatment and 1 per pen) were selected for evaluation of meat quality. The breast samples were divided into two equal halves. pH was measured in the right breast (m. pectoralis major) 24 h post-mortem using a portable pH meter with a temperature-compensated piercing electrode (HI 98163, Hanna instruments, Nușfalău, Romania), calibrated at pH 4.0 and 7.0 buffers. Color parameters (L*a*b*) were recorded on the same muscle 24 h post-mortem using a Minolta CR-330 chromameter (Konica Minolta, Tokyo, Japan), which was calibrated with a white plate according to the CIELAB system. Color and pH values represented the average of two repeated measurements per breast.

Drip loss (DL) was determined using fresh samples from the right breast (m. pectoralis minor). The samples were weighed, suspended with plastic bag cover, and stored at 4 °C for 24 h. After being dried with a paper towel, they were weighed again to calculate DL.

The left breast (m. pectoralis major) of the same birds was used to determine thawing and cooking loss (CL). The samples were placed in food-grade plastic bags and stored in a freezer at −20 °C for one month. Afterwards, the samples were thawed at 4 °C for 24 h, removed from the bags, blotted, and reweighed in order to calculate the thawing loss (TL). To determine CL, the samples were vacuum-sealed in food-grade plastic bags and cooked in a water bath set at 80 °C until the core temperature of the heaviest breast sample reached 74 °C. After cooking, samples were transferred to a tray, cooled to 20 °C at room temperature, gently blotted with paper towels and reweighed to calculate the cooking loss. Total loss was determined by combining the TL and CL.

Warner-Bratzler shear force (WBSF) was measured on cooked breast samples. Cores (1 cm^2^ pieces) were excised from the samples. WBSF was measured at three points per piece and represented the average of three repeated measurements using a TA.XT PLUS texture analyzer (Stable Micro Systems Ltd., Godalming, Surrey, UK), which was equipped with a Warner-Bratzler blade and a 50 kg load cell with a crosshead speed of 250 mm/min. The cuts were made perpendicular to the direction of the muscle fibers. Results were presented in kg.

To determine the nutritional composition, left thighs were deboned and the samples were ground and placed in a Petri dish. Water, protein, fat, collagen, and ash content were determined using near-infrared (NIR) spectroscopy with a DA6200 meat analyzer (PerkinElmer, Inc., Wellesley, MA, USA) with transmission spectroscopy using diode array detectors in the wavelength range of 850 to 1050 nm.

### 2.6. Blood Parameters

At the end of the trial (day 42), 5 mL of whole blood was collected from 14 birds per treatment into 9 mL serum-separator tubes (S-Monovette Serum Gel, Sarstedt AG & Co. KG, Nümbrecht, Germany). The serum was separated by centrifugation at 2500× *g* for 10 min, after which it was stored at −80 °C until analysis. The concentration of serum total protein, total cholesterol, HDL-cholesterol, LDL-cholesterol, and triglycerides were determined using commercial kits (Diagnosticum Ltd., Budapest, Hungary).

### 2.7. Intestinal Morphometry

The intestinal length of the same 70 birds that already used for the meat quality evaluation was also measured. The small intestine and ceca were excised and divided into three segments: the duodenum (from the ventriculus to the entry of the pancreatic and biliary ducts); the jejunum (from the entry of bile duct entry to Meckel’s diverticulum); and the ileum (from Meckel’s diverticulum to the ileocecal junction). The length of each segment and the ceca were recorded and expressed in cm per 100 g of live weight (LW). The total length of the small intestine was calculated as the sum of the three segments.

### 2.8. Statistical Analysis

Data were analyzed using the general linear model (PROC GLM) procedure of SAS (SAS^®^ OnDemand for Academics, version 3.81; SAS Institute Inc., Cary, NC, USA). BW was analyzed using analysis of covariance (ANCOVA) with the initial BW included as covariate. Phase-specific BWG were analyzed using ANCOVA, with BW at the beginning of each respective phase included as a covariate. Cumulative BWG (Day 1–42), FI, and FCR were subjected to one-way analysis of variance (ANOVA). Carcass traits, meat quality traits, blood parameters, and intestinal morphometrics were subjected to one-way ANOVA, with dietary treatment (Control, HI2, HI4, TM2, and TM4) as the fixed effect. After fitting each model, the normality of residuals was assessed using the Shapiro–Wilk test. For BW, BWG, FI and FCR, the experimental unit was the pen, and for carcass and meat physicochemical traits, blood and gut parameters, the experimental unit was the single carcass/bird. Least-squares means were compared using the Bonferroni correction, declaring significance at *p* < 0.05. Mortality data were analyzed using chi-squared tests following the procedure of Marascuilo [26].

## 3. Results and Discussion

### 3.1. Growth Performance

In the present study, including full-fat HI or TM at a level of up to 4% in broiler diets did not affect overall performance (BW, FI, FCR and mortality) (Table 3 and Table 4). On day 14, the TM4 and HI4 groups displayed the highest BW, whereas the HI2 and TM2 groups showed the lowest values, with the Control group remaining intermediate. By day 42, the TM4 group achieved a significantly higher final BW compared with HI2 (*p* < 0.05), while the Control, HI4, and TM2 groups presented intermediate values.

A comparable pattern was observed for BWG. During the D7–14 period, birds in the TM4 and HI4 groups showed greater BWG than those in the HI2 and TM2 groups, with the Control group displaying intermediate values (*p* < 0.001). During D35–42, BWG was higher in the TM4 group than in HI2, whereas TM2, HI4, and Control did not differ significantly from either group (*p* < 0.05). Over the entire 42-day experimental period, cumulative BWG did not differ among treatments (*p* > 0.05). Overall, these findings indicate that dietary inclusion of insect meals up to 4% did not compromise broiler growth performance throughout the 42-day rearing period. In consistent with previous studies on HI meal, which have also demonstrated that low inclusion levels do not impair the growth performance of broiler chickens [27,28]. Similar observations were made by Dabbou et al. [8] and Biasato et al. [27], who found that partially replacing soybean meal with 5% HI meal did not significantly affect production parameters. However, the responses to TM meal differed from those reported in previous studies. The BW, BWG, and FCR did not differ significantly among treatments (*p* > 0.05). Previous studies have demonstrated that the inclusion of 5% TM meal can significantly enhance the growth performance of broilers [27,29]. These discrepancies are likely due to differences in experimental design, insect rearing conditions, dietary formulation and the use of lower dietary inclusion levels across studies.

### 3.2. Carcass Traits

Although growth performance remained stable, the distribution of the carcass was significantly modified (Table 5). Compared to the Control group breast yields were higher in all insect-fed groups, representing an increase of approximately 1.6–2.3% (*p* < 0.001), a commercially relevant improvement. However, carcass yield, leg yield, and organ percentages remained unaffected (*p* > 0.05). In the case of HI, Schiavone et al. [28] and Biasato et al. [27] reported no significant changes in carcass yield at 5% inclusion level. For TM, Biasato et al. [29] observed no significant differences in carcass traits, although they noted a higher breast yield. However, Biasato et al. [27] found a significantly higher breast yield in broilers fed TM meal (at 5%), with no changes observed in other carcass parameters, which is consistent with the results obtained in the present study.

The selective increase in breast yield without compromising other carcass parameters is a notable finding with significant commercial implications. Since breast fillets are the valuable part of the carcass [30,31], this effect could offset the higher cost of insect-derived ingredients, improving the economic return and overall value of the final product. This likely results from preferential partitioning of limiting essential amino acids (lysine, methionine) toward breast muscle protein synthesis, as insect meals provide higher levels of these amino acids compared to corn-soybean diets [12]. This enhancement indicates species-specific metabolic effects of insect meals that warrant further investigation.

### 3.3. Meat Quality

The quality characteristics of breast meat were only marginally affected (Table 6). Color parameters, DL, TL, and WBSF in breast meat were unaffected (*p* > 0.05). However, birds fed the HI4 diet exhibited a slightly lower pH value than the Control group (5.77 vs. 5.89; *p* < 0.05), although values remained within acceptable ranges (pH 5.75–5.90) for broiler breast meat [28,29]. These birds also showed greater CL and total losses compared to the Control group (*p* < 0.05), indicating a moderately reduced water-holding capacity (WHC) at higher HI inclusion levels.

In meat science, the inverse relationship between lower muscle pH and reduced WHC is well-established. Reduced muscle pH promotes the dissociation of regulatory proteins from thick filaments via proton-induced mechanisms, leading to the unfolding and increased denaturation of myofibrillar proteins. This subsequently reduces the water-binding capacity of the muscle matrix [32]. In practical terms, the observed increase in CL (approx.1.9%) represents a modest yet measurable reduction in yield during thermal processing. Importantly, the observed cooking loss values remained within the generally accepted range for commercial processing of broiler breast meat [32], aligning with typical commercial performance expectations.

Protein content in thigh meat was significantly lower (*p* < 0.001) in the HI4 and TM2 groups than in the Control group, while HI2 and TM4 levels were intermediate (Table 7). Lipid content was significantly higher (*p* < 0.001) in all insect-fed groups compared to the Control group. These results indicate altered nutrient deposition, with dietary insect meal inclusion promoting lipid accumulation in meat, while reducing protein content modestly. These results suggest that full-fat insect meals promote fat deposition. This has already been confirmed for abdominal fat in broilers fed HI [33] and TM inclusion [34]. However, Biasato et al. [27] did not observe any significant changes to the proximate composition of thigh meat when broilers were fed diets containing 5% HI or TM meals. Conversely, Šťastník et al. [35] reported elevated fat levels in the thigh meat of broilers receiving a diet containing 5% TM meal. From a nutritional quality perspective, the modest increase in lipid content may improve the flavor and palatability of thigh meat. However, it represents a slight reduction in protein density compared to Control meat [28], which is consistent with the results of previous studies that examined the effects of insect meal on meat composition [27,33]. However, no sensory analyses were conducted in the present study.

### 3.4. Blood Parameters

From a broader perspective, blood biochemical indices are sensitive indicators of the birds’ metabolic status and organ function. Parameters such as total protein, cholesterol, and triglycerides are frequently used to diagnose metabolic disturbances in key organs [36]. Serum total protein content was higher in all insect-fed groups compared to the Control group (*p* < 0.001), suggesting enhanced protein metabolism (Table 8). This elevation indicates active protein synthesis and metabolism and may reflect the improved bioavailability of AA from the insect meals, particularly given their well-documented balanced profiles [27]. Triglyceride concentrations did not differ significantly among treatments (*p* > 0.05), consistent with the lack of differences in overall lipid metabolism across dietary treatments. These findings align with the observed lipid accumulation in thigh meat [33], likely reflecting efficient absorption of dietary lipids from the full-fat insect meals.

Total cholesterol levels were significantly higher in the TM-fed birds than in the Control and HI groups (*p* < 0.05). This elevation was driven by increases in high-density lipoprotein (HDL)-cholesterol (1.50–1.52 mmol/L in TM vs. 1.23–1.29 mmol/L in HI), while the Control group showed intermediate values (1.34 mmol/L). LDL-cholesterol levels remained unaffected by dietary treatments (*p* > 0.05). The shift towards the HDL fraction without a concurrent increase in LDL-cholesterol suggests favorable modulation of the lipid profile associated with the inclusion of TM meal.

In poultry, synthesis and secretion pathways of VLDL and HDL are similar, and those are the predominant circulating lipoproteins and serve as vehicles for lipid transport from liver to peripheral tissues [37]. Thus, the elevated HDL-cholesterol observed with TM meal likely reflects enhanced lipid mobilization to support rapid broiler growth rather than mammalian-style reverse cholesterol transport.

Based on the literature the FA composition differs markedly between the two insect species: HI larvae contain higher levels of saturated MCFAs, particularly C12:0 (6–8% of total FAs) and C16:0. In contrast, TM larvae contain higher levels of monounsaturated fatty acids (MUFAs) C18:1 (40% of total FAs) and polyunsaturated fatty acids (PUFAs) C18:2 (~15% of total FAs) [38,39]. These compositional differences are likely to explain the observed variations in blood lipid profiles between the two species. Previous studies reported no significant changes in the blood parameters of broiler chickens fed increasing levels (5–15%) of insect meal (HI or TM) [7,8,29]. In contrast, Jiang et al. [40] observed an increase in total cholesterol in TM-fed broilers, which is similar to the present study. This increase was only at inclusion levels of ≥9% and was not accompanied by a change in HDL concentration.

### 3.5. Intestinal Morphometry

The morphological characteristics of gut segments are well-established indicators of nutrient digestibility and overall absorptive capacity in poultry. It is widely recognized that the gastrointestinal tract (GIT) of birds exhibits dynamic morphometric adaptations in response to dietary nutrient availability [41].

Analysis of gut morphometry revealed that TM, but not HI, reduced small intestinal length (Table 9). Birds fed the TM4 diet had significantly shorter small intestines than those in the Control, HI2, and HI4 groups (*p* < 0.05). Intermediate values were observed in the TM2 group (6.04 cm/100 g BW), although this difference was not statistically significant (*p* > 0.05). Similarly, ileal length decreased in both the TM2 and TM4 groups compared to the Control, while the HI groups were unaffected. No significant differences were observed in duodenal, jejunal or cecal lengths between the different treatments (*p* > 0.05).

These localized, TM-specific effects on ileal morphometry contrast with the general lack of morphometric changes associated with HI inclusion, indicating species-specific impacts of insects on intestinal development. The findings regarding HI are consistent with previous reports indicating that dietary HI meal inclusion does not affect gut morphometry in chickens [42,43]. In contrast, the results obtained for TM differ from those of earlier studies. For example, Bovera et al. [44] reported that broilers fed TM meal as a full replacement for soybean meal had longer total, ileal, and cecal lengths, while Sedgh-Gooya et al. [45] found no morphometrical changes at lower inclusion levels (up to 5%). Also, Bovera et al. [46] found that replacing some of the soybean meal with HI larvae meal increased the lengths of the jejunum and the total intestine in laying hens.

Overall, discrepancies in intestinal length across studies may primarily reflect differences in feed digestibility and nutrient density of feeds. Diets with lower digestibility coefficients can promote compensatory intestinal elongation to increase the absorptive surface area [47,48]. Conversely, if the reduction in intestinal length reflects improved nutrient absorption efficiency due to the superior digestibility of full-fat TM meal, this would suggest that the insect meal, particularly its FA profile which is enriched in oleic and linoleic acids, enhances the efficiency of epithelial nutrient transport. Moreover, the present findings suggest that the reduced ileal length in TM-fed birds reflects improved nutrient digestibility and absorptive efficiency, meaning that less intestinal surface area is required to achieve adequate nutrient uptake. However, digestibility was not evaluated in the present study. Further experiments incorporating apparent ileal digestibility measurements are therefore required to clarify the underlying mechanisms and confirm this hypothesis.

## 4. Conclusions

Dietary inclusion of full-fat HI or TM meal at low levels (2–4%) did not impair overall broiler growth performance, feed efficiency, mortality, or carcass yield compared to the control diet. Both insect meals significantly increased breast meat yield relative to control, representing a commercially relevant improvement. Treatment-specific effects included reduced breast muscle pH and WHC in the HI4 group, and elevated serum HDL-cholesterol with shorter ileal length in TM-fed birds. All measured parameters remained within acceptable physiological ranges.

Direct comparison revealed no consistent overall advantage of either HI or TM over the other across growth performance, carcass traits, or meat quality parameters. Both insect meals demonstrated nutritional feasibility at these inclusion levels, with only subtle species-specific physiological responses. This study did not assess nutrient digestibility, which limits conclusions regarding feed utilization efficiency. Future research incorporating digestibility trials and economic analysis is needed to evaluate practical commercial viability. The present findings support low-level inclusion of full-fat HI or TM meal as sustainable protein sources for broiler nutrition under appropriate conditions.

## Figures and Tables

**Table 1:** Ingredients of the experimental diets (g/kg as fed).

	Treatments				
	Control	HI2	HI4	TM2	TM4
Experimental concentrate ^1^	850	850	850	850	850
Corn	59.5	66.0	68.0	68.0	76.0
Soybean meal	10.0	5.00	5.00	10.0	10.0
Full-fat soybean	76.0	56.0	36.0	48.0	20.0
Full-fat HI Meal	0.00	0.00	0.00	20.0	40.0
Full-fat TM meal	0.00	20.0	40.0	0.00	0.00
Calcium carbonate	3.00	1.50	0.00	3.50	4.00
Methionine	1.00	1.00	1.00	0.50	0.00
Valine	0.50	0.50	0.00	0.00	0.00

^1^ Concentrate based on corn, wheat, sunflower meal, soybean meal, full fat soybean, limestone, broiler grower complete premixture; HI: *Hermetia illucens*; TM: *Tenebrio molitor*.

**Table 2:** Nutritional composition, apparent metabolizable energy (AMEn), mineral, and amino acids content of the experimental diets.

	Treatments				
	Control	HI2	HI4	TM2	TM4
Analyzed, g/kg, as fed					
Dry matter	887	890	889	892	892
Crude protein	198	190	194	189	191
Sugar	49.0	49.0	47.0	47.0	46.0
Starch	416	430	435	433	444
Crude fiber	38.8	39.0	39.8	39.2	38.4
Fat	45.0	45.8	43.2	43.8	41.2
Ash	58.1	55.4	55.4	56.7	56.4
AMEn, MJ/kg	12.2	12.3	12.4	12.3	12.2
Calculated, %					
Available Ca	1.03	1.05	1.07	1.05	1.07
Available P	0.49	0.50	0.51	0.50	0.51
SID Lysine	1.14	1.14	1.15	1.14	1.14
SID Methionine	0.58	0.59	0.59	0.54	0.49
SID Methionine + Cystine	0.90	0.91	0.91	0.90	0.90
SID Threonine	0.75	0.75	0.76	0.76	0.77
SID Tryptophan	0.24	0.24	0.24	0.24	0.24
SID Arginine	1.18	1.16	1.17	1.17	1.16
SID Valine	0.89	0.91	0.89	0.88	0.91
SID Glycine + Serine	1.58	1.60	1.63	1.63	1.68

HI: *Hermetia illucens*; TM: *Tenebrio molitor*; SID: standard ileal digestibility.

**Table 3:** Effect of the dietary inclusion of either 0% (Control), 2% (HI2, TM2), or 4% (HI4, TM4) full-fat *Hermetia illucens* and *Tenebrio molitor* meals on broiler growth performance.

	Treatments					RSD ^1^	*p*-Value
	Control	HI2	HI4	TM2	TM4		
N. (birds)	350	350	350	350	350		
N. (pens)	14	14	14	14	14		
BW, g/bird ^^^							
Day 1	41.3	41.4	41.7	41.5	41.4	0.37	0.0898
Day 7	169	167	170	169	170	4.71	0.4394
Day 14	423 ^ab^	419 ^b^	427 ^a^	419 ^b^	429 ^a^	6.88	0.0005
Day 21	826	815	822	827	825	16.8	0.3567
Day 28	1304	1298	1319	1283	1286	44.7	0.2447
Day 35	1836	1856	1859	1862	1865	46.0	0.5393
Day 42	2411 ^ab^	2392 ^b^	2420 ^ab^	2425 ^ab^	2451 ^a^	44.4	0.0179
BW gain (BWG), g/d/bird ^#^							
D1–7	18.2	17.9	18.4	18.2	18.4	0.66	0.3822
D7–14	36.2 ^ab^	35.7 ^b^	36.8 ^a^	35.7 ^b^	37.1 ^a^	0.99	0.0007
D14–21	57.5	55.5	57.2	57.4	57.7	2.39	0.1424
D21–28	68.8	67.8	70.7	65.8	66.2	6.38	0.2491
D28–35	76.7	79.6	80.5	80.5	80.8	6.60	0.4294
D35–42	78.7 ^ab^	76.6 ^b^	81.4 ^ab^	81.9 ^ab^	85.5 ^a^	6.31	0.0066
D1–42	56.3	55.9	56.7	56.7	57.3	1.22	0.0597

^1^ Residual standard deviation; ^^^ BW covariate-adjusted LSMEANS using initial BW (Day 1) as covariate; ^#^ phase BWG = covariate-adjusted using BW at phase start; ^a,b^ Means in the same row with different superscript letters differ for *p* < 0.05.

**Table 4:** Effect of the dietary inclusion of either 0% (Control), 2% (HI2, TM2), or 4% (HI4, TM4) full-fat *Hermetia illucens* and *Tenebrio molitor* meals on broiler feed intake (FI), feed conversion ratio (FCR), and mortality.

	Treatments					RSD ^1^	*p*-Value
	Control	HI2	HI4	TM2	TM4		
N. (pens)	14	14	14	14	14		
FI, g/d/bird							
D1–7	21.3	21.2	21.3	21.1	21.1	0.40	0.6065
D7–14	43.7	42.8	43.4	43.3	43.1	1.41	0.4728
D14–21	82.5	82.9	82.7	83.1	84.0	3.48	0.8143
D21–28	137	136	135	135	135	5.98	0.9397
D28–35	145	144	150	147	147	6.22	0.1105
D35–42	189	182	182	178	182	22.9	0.8119
D1–42	103	101	102	101	102	3.78	0.7413
FCR							
D1–7	1.17	1.18	1.16	1.16	1.15	0.04	0.5053
D7–14	1.21	1.19	1.18	1.21	1.17	0.06	0.2184
D14–21	1.44	1.47	1.47	1.43	1.49	0.09	0.4674
D21–28	2.01	2.00	1.92	2.13	2.07	0.25	0.2341
D28–35	1.93	1.83	1.94	1.81	1.82	0.25	0.4696
D35–42	2.32	2.41	2.26	2.23	2.19	0.35	0.4687
D1–42	1.83	1.82	1.81	1.78	1.78	0.07	0.2832
Mortality, %	1.14	0.86	0.86	1.14	1.14	0.34 ^2^	0.9873 ^3^

^1^ Residual standard deviation; ^2^ Chi-square; ^3^ K-proportions test.

**Table 5:** Effect of the dietary inclusion of either 0% (Control), 2% (HI2, TM2), or 4% (HI4, TM4) full-fat *Hermetia illucens* and *Tenebrio molitor* meals on broiler carcass traits and yields.

	Treatments					RSD ^1^	*p*-Value
	Control	HI2	HI4	TM2	TM4		
N. (birds)	28	28	28	28	28		
Carcass, %SW ^2^	71.8	72.8	72.5	71.8	71.3	2.75	0.2547
Breast, %CW ^3^	27.6 ^b^	29.3 ^a^	29.9 ^a^	29.2 ^a^	29.4 ^a^	1.57	<0.0001
Leg, %CW	30.5	29.9	30.4	30.0	30.5	1.52	0.3958
Liver, %SW	1.53	1.54	1.46	1.49	1.50	0.25	0.7107
Gizzard, %SW	1.01	0.99	0.98	1.03	0.99	0.16	0.7767

^1^ Residual standard deviation; ^2^ Slaughter weight; ^3^ Carcass weight; ^a,b^ Means in the same row with different superscript letters differ for *p* < 0.05.

**Table 6:** Effect of the dietary inclusion of either 0% (Control), 2% (HI2, TM2), or 4% (HI4, TM4) full-fat *Hermetia illucens* and *Tenebrio molitor* meals on breast meat physical quality.

	Treatments					RSD ^1^	*p*-Value
	Control	HI2	HI4	TM2	TM4		
N.	14	14	14	14	14		
L*	62.8	63.2	62.7	62.8	64.6	2.40	0.2217
a*	12.2	11.6	12.5	12.2	11.5	1.39	0.2545
b*	12.6	13.6	12.9	13.9	13.8	1.63	0.1717
pH	5.89 ^a^	5.80 ^ab^	5.77 ^b^	5.84 ^ab^	5.81 ^ab^	0.09	0.0181
Drip loss, %	4.07	3.96	3.93	3.80	3.90	0.78	0.9276
Thawing loss, %	7.07	7.42	7.44	7.01	7.37	1.37	0.8652
Cooking loss, %	27.3 ^b^	28.4 ^ab^	29.2 ^a^	28.1 ^ab^	28.9 ^ab^	2.21	0.0215
Total loss, %	34.4 ^b^	35.8 ^ab^	36.7 ^a^	35.1 ^ab^	36.3 ^ab^	2.85	0.0418
WBSF ^2^, kg	1.67	1.58	1.59	1.64	1.63	0.31	0.2455

^1^ Residual standard deviation; ^2^ Warner-Bratzler shear force; ^a,b^ Means in the same row with different superscript letters differ for *p* < 0.05.

**Table 7:** Effect of the dietary inclusion of either 0% (Control), 2% (HI2, TM2), or 4% (HI4, TM4) full-fat *Hermetia illucens* and *Tenebrio molitor* meals on the proximate composition (g/100 g meat) of chicken thigh meat.

	Treatments					RSD ^1^	*p*-Value
	Control	HI2	HI4	TM2	TM4		
N.	14	14	14	14	14		
Water	72.9	72.5	72.7	73.1	72.9	0.58	0.0962
Protein	19.0 ^a^	18.7 ^ab^	18.5 ^b^	18.5 ^b^	18.8 ^ab^	0.31	<0.0001
Fat	4.67 ^b^	5.62 ^a^	5.92 ^a^	5.61 ^a^	5.32 ^a^	0.59	<0.0001
Collagen	1.03	1.06	1.06	1.05	1.02	0.08	0.4546
Ash	1.70	1.70	1.68	1.67	1.71	0.06	0.3813

^1^ Residual standard deviation; ^a,b^ Means in the same row with different superscript letters differ for *p* < 0.05.

**Table 8:** Effect of the dietary inclusion of either 0% (Control), 2% (HI2, TM2), or 4% (HI4, TM4) full-fat *Hermetia illucens* and *Tenebrio molitor* meals on serum biochemical parameters of broiler.

	Treatments					RSD ^1^	*p*-Value
	Control	HI2	HI4	TM2	TM4		
N.	14	14	14	14	14		
Total protein, g/L	24.4 ^b^	28.7 ^a^	28.1 ^a^	28.7 ^a^	29.2 ^a^	3.12	0.0009
Triglycerides, mmol/L	0.45	0.54	0.55	0.54	0.61	0.16	0.1350
Total Cholesterol, mmol/L	3.85 ^b^	3.75 ^b^	3.67 ^b^	4.29 ^a^	4.33 ^a^	0.36	0.0125
HDL ^2^, mmol/L	1.34 ^ab^	1.23 ^b^	1.29 ^b^	1.52 ^a^	1.50 ^a^	0.19	0.0003
LDL ^3^, mmol/L	1.26	1.31	1.18	1.25	1.30	0.29	0.7357

^1^ Residual standard deviation; ^2^ High density lipoproteins; ^3^ Low density lipoproteins; ^a,b^ Means in the same row with different superscript letters differ for *p* < 0.05.

**Table 9:** Effect of the dietary inclusion of either 0% (Control), 2% (HI2, TM2), or 4% (HI4, TM4) full-fat *Hermetia illucens* and *Tenebrio molitor* meals on intestinal morphometry (cm/100 g BW).

	Treatments					RSD ^1^	*p*-Value
	Control	HI2	HI4	TM2	TM4		
N.	14	14	14	14	14		
Small intestine	6.36 ^a^	6.33 ^a^	6.29 ^a^	6.04 ^ab^	5.73 ^b^	0.62	0.0485
Duodenum	0.95	0.96	0.99	1.01	0.92	0.11	0.2306
Jejunum	2.61	2.64	2.68	2.58	2.40	0.34	0.2601
Ileum	2.80 ^a^	2.72 ^ab^	2.62 ^ab^	2.45 ^b^	2.41 ^b^	0.35	0.0151
Cecum	0.71	0.67	0.66	0.70	0.63	0.08	0.0633

^1^ RSD: residual standard deviation; ^a,b^ Means in the same row with different superscript letters differ for *p* < 0.05.

## Data Availability

The raw data supporting the conclusions of this article will be made available by the authors on request.
